# Dissecting the natural phytochemical diversity of carrot roots with its colour using high performance liquid chromatography and UV–Visible spectrophotometry

**DOI:** 10.1016/j.heliyon.2024.e35918

**Published:** 2024-08-08

**Authors:** Acharya Balkrishna, Monali Joshi, Sarika Gupta, M. Priya Rani, Jyotish Srivastava, Pardeep Nain, Anurag Varshney

**Affiliations:** a Drug Discovery and Development Division, Patanjali Research Foundation, Governed By Patanjali Research Foundation Trust, NH-58, Haridwar, 249 405, Uttarakhand, India; b Department of Allied and Applied Sciences, University of Patanjali, Patanjali Yog Peeth, Roorkee, Haridwar Road, Haridwar, 249 405, Uttarakhand, India; c Patanjali Yog Peeth (UK) Trust, 40 Lambhill Street, Kinning Park, Glasgow, G411AU, UK; d Special Centre for Systems Medicine, Jawaharlal Nehru University, New Delhi, 110 067, India

**Keywords:** Anthocyanins, Carrots, *β*-Carotene, Chlorogenic acid, HPLC, Methyl caffeate

## Abstract

The research provides insights into the phytoconstituents of black, orange and red carrots (*Daucus carota* subsp. Sativus (Hoffm.) Schübl. & G. Martens), a highly nutritious food crop widely appreciated across age groups. Recognising carrots as a repository of health-promoting compounds, our study employs UV–Visible spectrophotometric and HPLC methods to discern significant variations in bioactive components among carrot varieties. Black carrots emerge as potent contenders, displaying the highest levels of total phenolics (2660 ± 2.29 mg GAE/100 g F W.), total flavonoids (831 ± 1.74 mg QE/100 g F W.), proanthocyanins (10910 ± 1.11 mg CE/100 g F W.), and tannins (713 ± 0.84 mg/100 g F W.). Red carrots, conversely, showcase higher anthocyanin content (6870 ± 1.85 mg CyGE/100 g F W.) by UV–Vis spectrophotometry. Additionally, orange carrots exhibit heightened *β*-carotene levels, confirmed at 0.03 μg/mg through HPLC. HPLC analysis unveils substantial chlorogenic acid variability (1.29 μg/mg) in black carrots, accompanied by the discovery of unique compounds such as cryptochlorogenic acid (0.05 μg/mg), caffeic acid (0.01 μg/mg), ferulic acid (0.11 μg/mg), methyl caffeate (0.01 μg/mg), and quercetin (0.02 μg/mg), marking the first detection of methyl caffeate in black carrots. The analytical methodology was meticulously validated encompassing optimal parameters such as linearity, precision, limit of detection, limit of quantification, accuracy, and robustness, within the range. In conclusion, our study underscores the health benefits of black carrots due to their rich polyphenolic content and endorses orange carrots for elevated *β*-carotene levels. These findings contribute to a deeper understanding of the diverse phytoconstituents in carrots, aid in informed dietary choices for improved health.

## Graphical abstract

**Figure:**
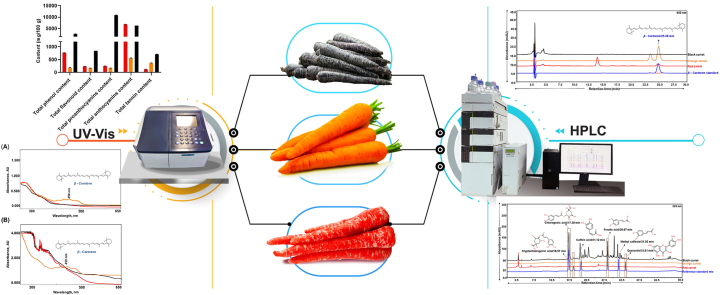

## Abbreviations

CAGR: Compound annual growth rate
HPLC: High performance liquid chromatography
HPTLC: High performance thin layer chromatography
UV: Ultra violet
*β*: Beta
DNA: Deoxyribonucleic acid
nm: Nanometer
ppm: Parts per million

## 1 Introduction

Carrot (*Daucus carota* subsp. Sativus (Hoffm.) Schübl. & G.Martens), a biennial herbaceous plant of Apiaceae family grown throughout the temperate zones across the world. The carrot holds significant economic importance as a vital food source, ranking among the top ten horticultural crops worldwide [1]. The global market for carrot is estimated to reach a compound annual growth rate (CAGR) of 4.10 % during the period 2024–2029 [2]. Carrots are found to be rich source of phenolic compounds such as chlorogenic acid, caffeic acid, 3′-caffeoylquinic acid, 4-*p*-coumaroylquinic acid, 3′,4′-dicaffeoylquinic acid, 3′,5′-dicaffeoylquinic, *p*-coumaric acid and ferulic acid, which have been widely recognised as natural antioxidants [3,4]. Carrots also exert substantial amount of therapeutically active phytoconstituents such as polyacetylenes (falcarinol, falcarindiol, falcarindiol-3-acetate), phenolics and carotenoids [5,6]. Polyacetylenes have been reported to have anti-inflammatory, antiproliferative, neurotoxic, anticancer, antiplatelet, antiallergic and antimicrobial activities [6,7].

Carrot roots are considered as the reservoir of major carotenes like *α*-carotene, *β*-carotene and *γ*-carotene, lycopene and *β*-zeacarotene [8]. They are majorly present not only in fruits, vegetables, algae and fungi but also in some animal derived products such as fish and egg yolk [9]. Based on the presence of natural pigments, carrots are divided into two groups: carotenoid rich carrots (*Daucus carota* L. ssp. *sativus*) responsible for red and orange color and anthocyanin rich carrots (*Daucus carota* L. ssp. *Sativus* var. atrorubens Alef.) responsible for purple color [10]. Carotenoids possess the ability to scavenge free radicals responsible for DNA damage, and inhibited the growth of tumour cells by interfering at diverse phases of cell cycle [11,12]. Anthocyanin belongs to the chemical class of flavonoids is considered bioactive, present in carrots, known to have nutritional values and effective in reducing different kinds of cancer [13,14]. Reports also showed the nutritional richness of carrots with carbohydrates, proteins, dietary fibres, fats, vitamins (for example, A, E, C, thiamin, riboflavin and niacin) and minerals (Ca, P, K, Na, Mg, Fe). Moreover, carrots enriched with vitamin A is considered to be an essential nutrient for healthy eye vision [15]. The major fatty acid present in carrot seed oil after supercritical carbon dioxide extraction was identified as oleic acid [16].

Previous studies have demonstrated that genotypes and environmental conditions significantly influence the content of phytoconstituents and sugars in plants. Warmer temperatures, for instance, can elevate terpene levels in carrot roots, imparting bitterness, whereas cooler conditions generally yield higher-quality crops [17]. Earlier research also highlighted black carrots as particularly rich in polyphenols, including hydroxycinnamic acid derivatives such as chlorogenic acid, 3-*O*-feruloylquinic acid, and *p*-coumaroyl quinic acid, positioning them as one of the foremost sources of anthocyanins [18].

Despite carrots being widely consumed and available in various colours, comprehensive comparative studies on phenolic content and other bioactive compounds across different root colours are notably lacking. This study aims to address this gap by focusing on red, orange, and particularly black carrot varieties cultivated at Haridwar, Uttarakhand, India. It explores the determination of total phenols, total flavonoids, total proanthocyanins, anthocyanins, and tannins. Additionally, the study provides detailed profiling of phenolic constituents and *β*-carotene using HPLC and UV–Vis spectrophotometry, emphasising significant differences in bioactive compounds attributed to black carrot varieties. The novelty of this study is the identification of methyl caffeate in black carrots, marking a pivotal advancement in our understanding, due to its reported plethora of pharmacological activities including antihyperglycemic, antidiabetic, anticancer, antimicrobial, and antiinflammatory [[19], [20], [21]]. This finding represents the first documented identification of methyl caffeate in black carrots through HPLC analysis.

## 2 Materials and methods

### 2.1 Reagents used

HPLC grade methanol (Cat # R047H21), tetrahydrofuran (Cat # R243E21), orthophosphoric acid (Cat # 00050) and dichloromethane (Cat # R031B21) were purchased from Rankem (India) and LCMS grade acetonitrile (Cat # 34967) was procured from Honeywell (Germany). Chlorogenic acid (Cat # 7329420) and quercetin (Cat # 7192377) were purchased from Sisco Research Laboratory (India). Cryptochlorogenic acid (Cat # CFS201902) from Chem Faces (China), catechin (Cat # C1251) from Sigma Aldrich (USA), ferulic acid (Cat # T15L143) from Natural Remedies (India), methyl caffeate (Cat # AY5AD), gallic acid (Cat # 91215) and *β*-carotene (Cat # HNBC20A) from Tokyo Chemical Industry (Japan) were also used. Cyanidin-3-*O*-glucoside (Cat # 16406) was purchased from Cayman (United States), Folin-Ciocalteu reagent (Cat # 0387000250) and potassium permanganate (Cat # 054100050) from Loba Chemie (India), aluminium chloride and vanillin (Cat # 61,860,301,001,730) from Merck (Germany) were also purchased.

### 2.2 Plant material

Different varieties of carrots - red, orange, and black (2 kg each) were purchased from a local market near carrot cultivation sites at Haridwar, Uttarakhand, India, during February 2021.These carrot samples were then verified by the in-house taxonomist of Patanjali Research Foundation Herbarium, Haridwar, Uttarakhand (Voucher number: 5938).

### 2.3 Extraction method

Around 500 g fresh and thoroughly cleaned carrots were grinded properly in a mixer grinder, and extracted separately with methanol for 3 h at 50 °C with continuous stirring in 3 cycles followed by filtration. After extraction, all the extracts were combined individually and concentrated under vacuum.

### 2.4 Determination of total phenolics

The total phenolic content of different carrot samples was determined by the Folin - Ciocalteu colorimetric method. Briefly, aliquots 1 mL stock solution of methanolic extracts was mixed with 1 mL of 10 % Folin - Ciocalteu reagent and incubated at room temperature for 5 min. The mixture was neutralised by adding 1 mL sodium carbonate (10 %) and kept in dark at room temperature for 1 h. The absorbance was measured at 760 nm against a blank on a UV–Vis spectrophotometer. Gallic acid was chosen as the standard. Total phenolic content was determined based on the standard curve plotted for gallic acid [22]. The data were expressed as mg gallic acid equivalent per 100 g of fresh weight (mg GAE per 100 g F. W.).

### 2.5 Determination of total flavonoids

Total flavonoid content was determined accordingly as aluminium chloride colorimetric method described by Chang et al. [23]. Aliquot 1 mL of the stock solution of extracts was mixed with 0.4 mL of 10 % aluminium chloride, 0.4 mL of 3 M sodium acetate and 3 mL of ethanol. After incubation at room temperature for 30 min, absorbance of the reaction mixture was measured at 450 nm on a UV–Vis spectrophotometer. Quercetin was taken as the standard. Total flavonoid content was determined based on the standard curve plotted for quercetin and the results were expressed as mg quercetin equivalent per 100 g of fresh weight (mg QE per 100 g F. W.).

### 2.6 Determination of proanthocyanins

Proanthocyanin content in the extracts was determined by using vanillin - hydrochloric acid assay. To 500 μL extract, 3 mL 4 % vanillin prepared in methanol was added along with 1.5 mL concentrated hydrochloric acid with vigorous stirring. After stirring, incubate the mixture at room temperature for 15 min and the absorbance of the mixture was measured at 500 nm. Catechin was used as the standard. The proanthocyanin content was expressed as mg catechin equivalent per 100 g of fresh weight (mg CE per 100 g F. W.) [24].

### 2.7 Determination of anthocyanins

The anthocyanin content in the samples was determined by incubating 4 mL of sample with 1 mL of 1 % acidified methanol overnight. After incubation, the reaction mixture was supplemented with 200 μL distilled water and 500 μL chloroform, and the absorbance was measured at 530 nm. Anthocyanin content was quantified using a standard curve prepared with cyanidin-3-*O*-glucoside [25] and expressed as mg cyanidin-3-*O*-glucoside equivalent per 100 g of fresh weight (mg CyGE per 100 g F. W.).

### 2.8 Determination of tannins

Tannin content was determined using titrimetric method, in which 1 g of extract was added with 100 mL of distilled water and stirred for 30 min. Filtered the solution using Whatman number 41 filter paper and aliquot 10 mL of the filtrate was mixed with 750 mL distilled water. Titrate the solution with 0.02 M potassium permanganate solution. The end point was the appearance of yellow colour to the solution. Distilled water was used as the blank. Titration was continued until concordant values were observed. The composition of tannins was calculated by using the formula,

Composition of tannins (mg/g) = (A-B) x M x 0.4157 x 100/W

where, A - Titre value of sample; B - Titre value of blank; M − Molarity; and W - Weight of extract taken.

### 2.9 High performance liquid chromatography method for the identification, validation and quantification of β-carotene and phenolic constituents from staple varieties of carrots

#### 2.9.1 Identification of β-carotene and phenolic constituents from staple varieties of carrots using HPLC method

##### 2.9.1.1 Preparation of standard solution

Stock solutions (1000 ppm) were prepared by dissolving accurately weighed standards (chlorogenic acid, cryptochlorogenic acid, caffeic acid, ferulic acid, methyl caffeate, and quercetin) in methanol and *β*-carotene in tetrahydrofuran. The stock solutions of phenolic standards were further diluted with methanol while *β*-carotene was diluted with tetrahydrofuran to make working standard solutions of concentration 50 ppm. The solutions were filtered using 0.45 μm nylon filter and was used for the analysis.

##### 2.9.1.2 Preparation of sample solution

Sample solutions were prepared by dissolving 0.25 g methanol extract of each carrot variety in 5 mL methanol separately and sonicated for 30 min. Filtered the solution using 0.45 μm nylon filter and was used for the analysis. For the estimation of *β*-carotene, tetrahydrofuran was used to dilute the methanol extracts of carrots.

##### 2.9.1.3 Instrumentation used and chromatographic conditions

HPLC analysis was performed using a modular Prominence-i HPLC system (LC-2030C 3D Plus, Shimadzu, Japan) comprising a pump, an autosampler, and a diode array detector. Chromatographic separation was conducted on a Shodex-C18 column (5 μm, 4.6 × 250 mm), (P21Q5027, Japan). LabSolutions software (version 6.92) was utilised for instrument control and data analysis.

For analysis of *β*-carotene, isocratic mode of elution was performed with acetonitrile: dichloromethane: methanol (70: 20: 10 v/v/v) as mobile phase using VDSphur C18-4E (250 mm × 4.6 mm x 5 μm) column. The detection was performed at 450 nm. While a gradient elution of 0.1 % orthophosphoric acid in water (pH adjusted to 2.5 with diethylamine) (solvent A) and acetonitrile (solvent B) were used as the mobile phase for the analysis of phenolic compounds. Total run time was 50 min and the concentration gradient varies as: 5 % B for 0–5 min, 10 % B for 5–10 min, 10–15 % B for 10–20 min, 15–40 % B for 20–30 min, 40–50 % B for 30–40 min, 50–70 % B for 40–45 min, 70 to 5 % B for 45–46 min and 5 % B for 46–50 min. A constant flow rate of 1 mL/min and 35 °C temperature was maintained throughout the method. The detection was performed at 325 nm for phenolic compounds. The compounds were identified using the level 1 identification method following the guidelines of the Metabolomics Standards Initiative (MSI) [26]. Level 1 identification entails comparing the metabolite with a reference standard. Structural files were sourced from ChemSpider (www.chemspider.com).

#### 2.9.2 Validation of HPLC parameters for the quantification of β-carotene and phenolic constituents in methanol extract of carrot

High performance liquid Chromatographic method for the quantification of *β*-carotene and phenolic constituents in carrots were validated in accordance with ICH Q2 (R1) guidelines [27].

##### 2.9.2.1 System suitability and specificity

System suitability parameters for HPLC analysis were established based on the percentage relative standard deviation (% RSD) of peak areas, tailing factor, and theoretical plates observed from six replicate injections (*n* = 6) of the standard solution. Specificity was assessed by injecting 10 μL of the solvent blank to ensure there was no interference with analyte peaks.

##### 2.9.2.2 LOD, LOQ and linearity

Limits of detection (LOD) and quantification (LOQ) were calculated using the signal-to-noise (S/N) ratio and % RSD of peak area. The S/N ratio for LOD and LOQ was verified by injecting six replicates of the component's least concentration at which it could be reliably detected and quantified. The % RSD limits for LOD and LOQ were set at not more than 33 % and 10 %, respectively. Linearity was determined by injecting various concentrations of the standard solution, resulting in a calibration curve with a correlation coefficient (*r*) exceeding 0.999.

##### 2.9.2.3 Precision and accuracy

System precision was performed on the basis of % RSD of the area obtained from 6 replicates injection of working standard solution. The % RSD value for system precision was set not more than (NMT) 2 %. Method precision was assessed based on the % RSD of the area obtained from a single injection of 6 replicate sample preparations. The criterion for method precision was established as a % RSD value of not more than 2 %. Intermediate precision of the method was assessed using % RSD values from six replicate injections (*n* = 6) of solutions on first day and repeat analysis on the second day with 6 replications (*n* = 6). Accuracy was evaluated through a recovery study of analysis, involving spiking reference standards into samples at known concentrations ranging from 80 %, 100 % and 120 %. Percent recovery at these levels was calculated by comparing the accepted reference value with the observed value.

##### 2.9.2.4 Range

The range of an analytical procedure is defined as the interval between the upper and lower concentrations of analyte in a sample for which the analytical procedure exhibits appropriate levels of precision, accuracy, and linearity. The quantification range for the current method was established to be between 6 and 10000 μg/g for *β-*carotene and 6 and 2000 μg/g for phenolic constituents. The range was derived from the calibration curve's linearity assessment, which demonstrated that the method is reliable and accurate across this concentration span.

##### 2.9.2.5 Robustnes*s*

The robustness of the method was evaluated by incorporating some intentional variations in the chromatographic conditions like flow rate, temperature, and change of column. For the present study, experiments were performed by varying the flow rate from 1 mL/min to 0.95 and 1.05 mL/min, and column temperature was modified to 33 °C and 37 °C from 35 °C at LOQ level for the validation of phenolic constituents; whereas flow rate and change of column was varied for the validation of *β-*carotene.

#### 2.9.3 High performance liquid chromatography method for the quantification of β-carotene and phenolic constituents from staple varieties of carrots

The quantitative assessment was carried out by calculating the response of phytoconstituents present in extracts in comparison with the reference standards under the same validated experimental conditions.

### 2.10 UV–visible spectrophotometric method for the detection of β-carotene in different varieties of carrots

#### 2.10.1 Preparation of sample solution and analysis

Sample stock solutions were prepared by dissolving 0.25 g methanol extract of each carrot variety in 3 mL methanol separately and sonicated for 30 min. The stock solutions were diluted 20 times and 80 times for recording different spectra for red and orange carrots; while in black carrots, the stock solution was diluted 50 times and 200 times for analysis. The solutions were filtered using 0.45 μm nylon filter and was used for recording UV–Vis spectra using UV–Vis spectrophotometer (UV-1800, Shimadzu, Japan), at a wavelength of 200–800 nm.

### 2.11 Statistical analysis

Data were reported as mean ± SD for triplicate measurements. Method validation was confirmed using a single-factor analysis of variance (ANOVA) to evaluate the linearity of the proposed method, performed with GraphPad Prism 8 software (GraphPad, San Diego, CA, USA). Statistical significance was considered at a *p*-value <0.001, within a 95 % confidence interval.

## 3 Results and discussion

### 3.1 Quantification of polyphenol contents in methanol extract of different varieties of carrots by UV–Vis spectrophotometric method

**Fig. 1:**
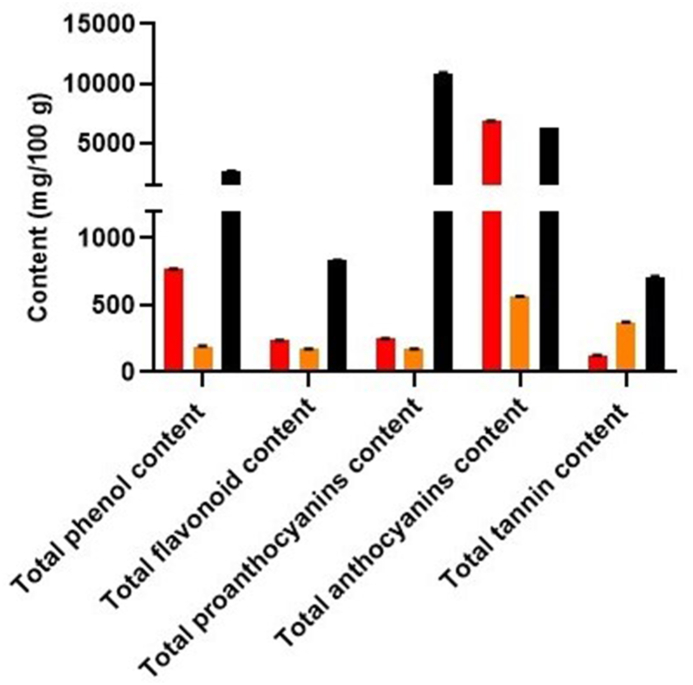
Determination of polyphenol contents in methanol extracts of red, orange and black carrot varieties using UV–Vis spectrophotometer. Methanol extract of black carrots showed highest content of total phenolics, flavonoids, proanthocyanins and tannins compared to red and orange carrots, while anthocyanin content was found to be high in red followed by black and orange carrots.

Polyphenol contents in various carrot varieties were determined spectrophotometrically. The methanol extract of black carrots exhibited the highest total phenol (2660 ± 2.29 mg GAE/100 g) and flavonoid content (831 ± 1.74 mg QE/100 g), followed by red carrots (770 ± 1.2 mg GAE/100 g of total phenol and 234 ± 2.67 mg QE/100 g of total flavonoid contents) and orange carrots (192 ± 2.76 mg GAE/100 g of total phenol and 170 ± 4.11 mg QE/100 g of total flavonoid contents). Proanthocyanin content varied among the varieties as follows: black carrots (10910 ± 1.11 mg CE/100 g) > red carrots (251 ± 1.85 mg CE/100 g) > orange carrots (172 ± 0.58 mg CE/100 g). Anthocyanin content was notably high in red (6870 ± 1.85 mg CyGE/100 g), followed closely by black (6372 ± 1.6 mg CyGE/100 g) and orange carrots (563 ± 2.63 mg CyGE/100 g)). Total tannin content followed a similar trend, with values of 713 ± 0.84 mg/100g, 371 ± 2.21 mg/100 g, and 124 ± 2.13 mg/100 g for black, orange, and red carrots respectively (Fig. 1).

Considering polyphenolic constituents as crucial antioxidant molecules [28], black carrots and red carrots might demonstrate significant antioxidant properties due to their high levels of total phenolics, total flavonoids, proanthocyanins, anthocyanins, and tannin contents. To conduct a more detailed analysis of the diverse bioactive compounds in these carrot varieties, an HPLC method was employed.

### 3.2 HPLC method for the identification, validation and quantification of β-carotene and phenolic compounds in methanol extract of different carrot varieties

**Fig. 2:**
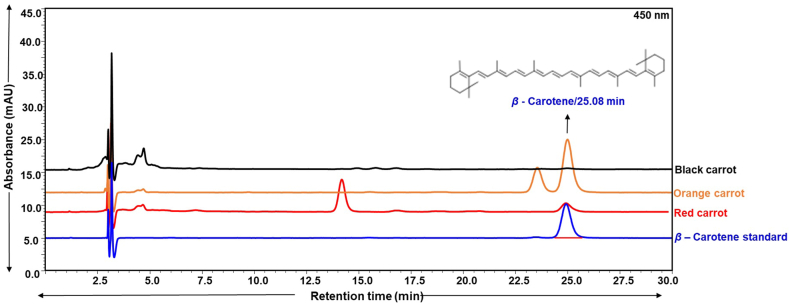
HPLC overlay chromatogram of standard *β*-carotene with extracts of black, orange and red carrots. The peak corresponds to *β*-carotene was observed at a retention time 25.08 min using isocratic elution with acetonitrile: dichloromethane: methanol (70: 20: 10 v/v/v) as mobile phase. The detection was performed at 450 nm. Blue colour indicates standard *β*-carotene, black colour indicates methanol extract of black carrots, orange colour indicates methanol extract of orange carrots and red colour indicates methanol extract of red carrots. Intense peak corresponds to methanol extract of orange carrots indicates the presence of highest content of *β*-carotene as given in Table 2. *β*-Carotene structure has been sourced from www.chemspider.com (Accessed on March 24, 2024).

High performance liquid chromatography was instrumental in discerning the presence of phenolic compounds in carrot by meticulously comparing their retention times against well-established standards [29]. The most accurate and reliable high performance liquid chromatography method was used to analyse and quantify the amount of *β*-carotene present in three staple varieties of carrots. In the optimised conditions, *β*-carotene was eluted at a retention time of 25.08 min (Fig. 2) and phenolic constituents including chlorogenic acid, cryptochlorogenic acid, caffeic acid, ferulic acid, methyl caffeate, and quercetin at 17.58, 18.97, 21.12, 28.67, 31.82 and 33.81 min respectively.

#### 3.2.1 High performance liquid chromatography – method validation

HPLC method used for the quantification of *β*-carotene and phenolic constituents *viz*., chlorogenic acid, cryptochlorogenic acid, caffeic acid, ferulic acid, methyl caffeate, and quercetin in methanol extract of carrot was validated as per ICH Q2 (R1) guidelines [27] in terms of system suitability, specificity, linearity, precision, limit of detection, limit of quantification, accuracy and robustness (Table 1).

**Table 1:** HPLC method validation for the quantification of *β*-carotene and phenolic constituents from methanol extract of carrot.

Sl.No.	Parameters	Acceptance criteria	*β*-Carotene	Chlorogenic acid	Cryptochlorogenic acid	Caffeic acid	Ferulic acid	Methyl caffeate	Quercetin
**1**	System Suitability	% RSD of Retention time - NMT 2 %	1.59	0.12	0.10	0.09	0.03	0.02	0.02
		% RSD of area - NMT 2 %	0.43	1.19	1.20	1.19	1.25	0.6	0.6
		USP tailing -NMT 2 %	0.98	1.35	1.37	1.39	1.36	1.29	1.39
		Theoretical plates – NLT 2000	29293	48816	54828	49210	206082	304000	342770
**2**	Specificity	No interference at retention time	Complies	Complies	Complies	Complies	Complies	Complies	Complies
		Peak purity	Complies	Complies	Complies	Complies	Complies	Complies	Complies
**3**	Linearity	Correlation coefficient NLT 0.99	1.0000	0.9993	0.9993	0.9994	0.9994	0.9994	0.9994
**4**	Precision								
	System	% RSD of area – NMT 2 %	0.43	0.85	0.87	0.87	0.92	0.92	0.89
	Intermediate	% RSD of assay – NMT 10 %	9.66	9.86	7.06	1.67	4.44	4.43	2.53
	Method	% RSD of assay – NMT 2 %	0.30	0.88	0.98	0.56	0.41	1.48	1.14
**5**	Limit of detection	S/N – NLT 3:1	6.12	5.19	6.86	6.21	9.29	6.51	5.16
		% RSD of area – NMT 33 %	3.91	4.08	1.57	1.33	0.82	1.71	1.88
**6**	Limit of Quantification	S/N – NLT 10:1	16.15	18.03	24.31	12.42	18.59	16.32	18.20
		% RSD of area – NMT 10 %	1.32	1.73	0.99	1.34	0.82	1.40	5.21
**7**	Accuracy	80 %–120 %	94.97–100.93 %	91.73–100.63 %	94.06–102.48 %	93.77–100.19 %	94.28–100.94 %	91.42–99.95 %	99.42 to 99.41 %
**8**	Range (μg/g)		6 to 10000	6 to 2000	6 to 2000	6 to 2000	6 to 2000	6 to 2000	6 to 2000
**9**	Robustness								
	Flow rate	% RSD of 18 determinations – NMT 20 %	9.70	9.15	10.74	1.76	7.69	10.90	10.07
	Temperature	% RSD of 18 determinations – NMT 20 %	NA	2.27	7.74	12.66	14.73	2.49	2.86
	Change of column	% RSD of 12 determinations – NMT 20 %	6.19	NA	NA	NA	NA	NA	NA

Note: All the parameters were validated as per the guidelines of ICH Q2 (R1). Abbreviations used: NMT, not more than; NLT, not less than; NA, not applicable.

##### 3.2.1.1 System suitability

The system suitability criteria for high-performance liquid chromatography (HPLC) were established by evaluating several parameters from multiple injections of standard solutions (*n* = 6). The assessment included the percent relative standard deviation (% RSD) of peak areas, tailing factor, and theoretical plates. These results demonstrate that the developed methods for *β*-carotene and phenolic constituents met the suitability requirements (Table 1).

##### 3.2.1.2 Specificity

The specificity of the HPLC method was assessed by examining any interference at the retention time of the analyte molecules. Validation of the retention time was performed by comparing it with reference standards during the analysis, which demonstrated no interference corresponding to the analyte peak in the solvent blank.

##### 3.2.1.3 Linearity and range

The linearity of *β*-carotene and phenolic constituents was assessed by injecting various concentrations of the standard solution, prepared by diluting standard stock solutions. The HPLC methods demonstrated a linear correlation with reference standards, indicated by correlation coefficients (*r*) exceeding 0.999. This confirms the method's linearity throughout the tested concentration ranges.

(refer to Table 1).

##### 3.2.1.4 LOD and LOQ

The determination of the limit of detection (LOD) and limit of quantification (LOQ) for *β*-carotene and phenolic constituents was based on the signal-to-noise ratio (S/N) and percent relative standard deviation (% RSD) of peak area at the LOD and LOQ levels. The LOD signal-to-noise ratio was established by injecting six replicates (*n* = 6) of the minimum concentration at which the analyte can be consistently detected. Similarly, the LOQ signal-to-noise ratio was determined by injecting six replicates (*n* = 6) of the concentration at which the analyte can be reliably quantified. In the context of high-performance liquid chromatography (HPLC), the maximum acceptable % RSD of peak area was set at 33 % for LOD and 10 % for LOQ (Table 1).

##### 3.2.1.5 Precision and accuracy

The precision of the analytical methods for quantifying *β*-carotene and phenolic constituents was evaluated through system, intermediate, and method analyses. The % RSD values obtained from HPLC were compared against acceptance criteria as detailed in Table 1, and were found to fall within the acceptable range. Therefore, the methods demonstrate high precision for the quantification of *β*-carotene, chlorogenic acid, cryptochlorogenic acid, caffeic acid, ferulic acid, methyl caffeate, and quercetin.

Accuracy was assessed through a recovery study using the standard addition method. Three different known concentration levels (80 %, 100 %, and 120 % of the expected concentration) were spiked into the sample. The recovery of analyte molecules ranged from 91 % to 103 %, indicating satisfactory accuracy. These results affirm that the precision and accuracy values for quantifying analyte molecules in the methanol extract of carrot met the acceptance criteria outlined in Table 1. This underscores the reliability and suitability of the methods for their intended purpose.

##### 3.2.1.6 Robustness

The robustness of an analytical method measures its ability to withstand minor variations in method parameters without significant impact on the measured values. In this study, variations in flow rate, column temperature, and type of column were deliberately introduced to assess their effect on recovery studies. It was observed that these parameter variations did not cause significant changes in the measured values. The % RSD of determinations was evaluated and found to be within permissible limits for variations in flow rate (from 1 mL/min to 0.95 mL/min and 1.05 mL/min) and column temperature (from 35 °C to 33 °C and 37 °C) for phenolic constituents. Additionally, two different types of columns were tested: VDSphur PUR 120 C18–U (250 mm × 4.6 mm, 5 μm) and Shodex C18-E (250 mm × 4.6 mm, 5 μm) for the validation of *β*-carotene. The % RSD of replicate analyses and mean recovery were obtained (refer to Table 1) to demonstrate the robustness of the method. These results confirm that the method is robust, as variations in flow rate, column temperature, and column type did not significantly affect the precision or recovery of analyte measurements. This underscores the reliability and consistency of the method under varying experimental conditions.

#### 3.2.2 Quantitative analysis of β-carotene and phenolic constituents in staple varieties of carrots

The quantitative analysis showed that maximum content of *β*-carotene was present in orange carrots accounted for 0.03 μg/mg, followed by red (0.01 μg/mg) and black carrots (≤0.01 μg/mg) (Table 2). Among the various carotenoids found in carrots, α- and *β*-carotene take precedence as the most abundant, imparting characteristic orange, red, and yellow hues to the vegetables [30]. In this study, the identification of maximum *β*-carotene content in orange carrots aligns seamlessly with earlier research, reinforcing the established knowledge that orange carrots contain approximately 20 % more carotenoids than other cultivars. Conversely, black/purple carrots were found to have the least amount of *β*-carotene [[31], [32]].

**Table 2:** Quantification of *β*-carotene and polyphenolic compounds in staple varieties of carrots using HPLC method with reference to the chromatograms given in Figs. 2 and 4.

S. No.	Compounds		Content (μg/mg F. W.)	
		Red carrot	Orange carrot	Black carrot
1	*β*-Carotene	0.01	0.03	≤0.01
2	Chlorogenic acid	0.03	0.23	1.29
3	Cryptochlorogenic acid	≤0.01	≤0.01	0.05
4	Caffeic acid	≤0.01	≤0.01	0.01
5	Ferulic acid	≤0.01	≤0.01	0.11
6	Methyl caffeate	≤0.01	≤0.01	1.1
7	Quercetin	≤0.01	≤0.01	1.2

**Fig. 3:**
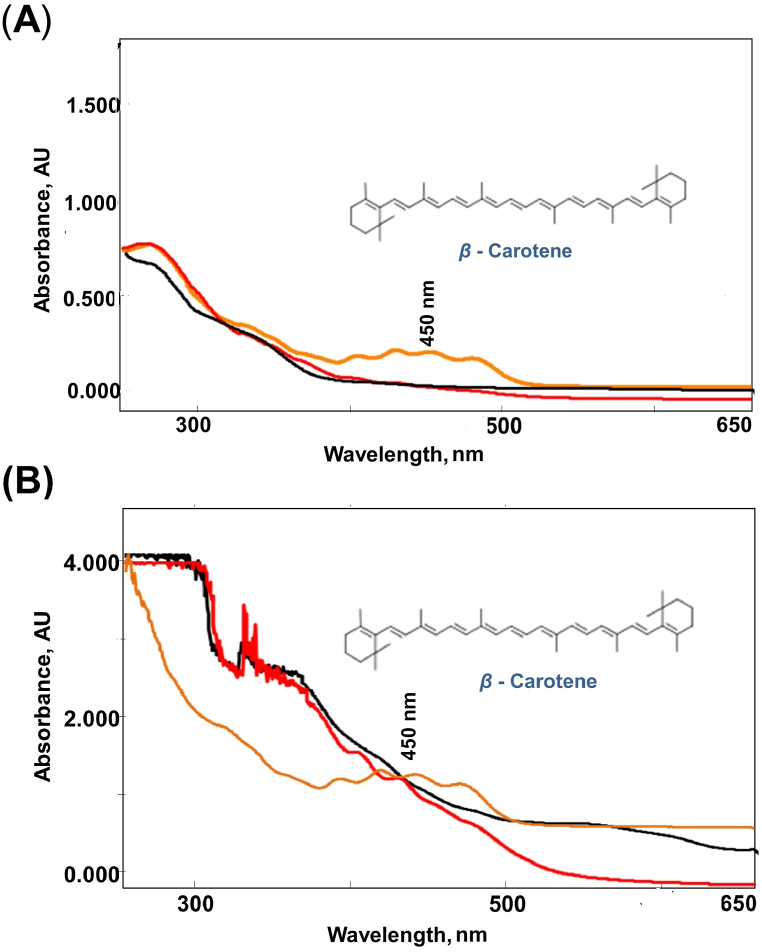
UV–Visible spectra of red, orange, and black carrots analysed using UV–Vis spectrophotometer (UV-1800, Shimadzu, Japan). In the spectra, x-axis denotes the range of wavelength (nm) and y-axis denotes the absorbance (AU). (**A**) denotes the overlay UV–Visible spectra of diluted solutions of extracts of red, orange, and black carrots and (**B**) denotes the overlay UV–Visible spectra of concentrated solutions of extracts of red, orange, and black carrots carrots. Represents red carrots as red, orange carrots as orange and black carrots as black. Orange carrots showed maximum absorbance in the visible region (450 nm) compared to red and black varieties. *β*-Carotene structure has been sourced from www.chemspider.com (Accessed on March 24, 2024).

Verification of these findings through UV–Vis spectrum analysis of orange carrots reveals maximum absorbance in the visible region (450 nm), confirming the presence of *β*-carotene (Fig. 3) compared to red and black carrots. *β*-Carotene, known as the precursor of vitamin A, is recognised for its efficient reduction of free radicals and potent antioxidant activity [33]. Highlighting the significance of *β*-carotene, studies have shown that *β*-carotene derived from natural sources exhibits excellent cytotoxic activity against leukaemia cells. At a dose of 20 μM, it arrests the cell cycle in the G1 phase and induces apoptosis, showcasing its potential in therapeutic applications [34]. A study was reported recently on the effects of tube and julienne shaped carrots and its oral processing behaviour on bolus properties and bioaccessibility of *β*-carotene in raw carrots [35]. These findings underscore the importance of *β*-carotene, not only in providing colour to carrots but also in offering potential health benefits through its antioxidant and cytotoxic properties.

**Fig. 4:**
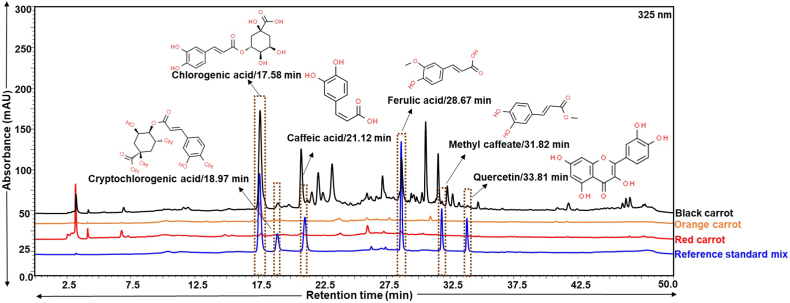
HPLC overlay chromatogram of phenolic standards in the form of a reference standard mix (chlorogenic acid (17.58 min), cryptochlorogenic acid (18.97 min), caffeic acid (21.12 min), ferulic acid (28.67 min), methyl caffeate (31.82 min), and quercetin (33.81 min)) with methanol extracts of different carrot varieties at 325 nm using 0.1 % orthophosphoric acid in water (pH 2.5) (solvent A) and acetonitrile (solvent B) as mobile phase. Methanol extract of black carrots showed enriched content of phenolic constituents as given in Table 2. Represents standard mix by blue colour, methanol extract of red carrots as red, orange carrots as orange and black carrots as black. Phytoconstituent structures have been sourced from www.chemspider.com (Accessed on March 24, 2024).

Aiming for optimal resolution of phenolic compounds, diverse solvent systems were explored to achieve the meticulous separation of constituents. Ultimately, an exemplary resolution was achieved through binary gradient elution employing 0.1 % orthophosphoric acid in water (pH 2.5) as solvent A and acetonitrile as solvent B. This dynamic mobile phase was employed for the in-depth analysis of phenolic compounds. Chlorogenic acid, exhibited a distinct elution peak at 17.58 min. Strikingly, the highest concentration was unveiled in black carrots (1.29 μg/mg), surpassing orange (0.23 μg/mg) and red (0.03 μg/mg) varieties. Furthermore, exclusive identification of other phenolic compounds, namely cryptochlorogenic acid (0.05 μg/mg), caffeic acid (0.01 μg/mg), ferulic acid (0.11 μg/mg), methyl caffeate (0.01 μg/mg), and quercetin (0.02 μg/mg), were confined to black carrots. These compounds manifested at retention times of 18.97, 21.12, 28.67, 31.82, and 33.81 min, respectively, as illustrated in Fig. 4.

Moreover, in the current investigation, chlorogenic acid stood out as the most prevalent among the examined phenolic compounds, aligning with prior research highlights of its antiviral, antibacterial, neuroprotective, cardio protective, and antitumor attributes [[3], [13], [36]]. Confirmation of the presence of phenolic compounds such as quercetin and ferulic acid in black carrots echoes the earlier findings in literature [37]. Moreover, this study marks the pioneer detection of methyl caffeate, the methyl ester of caffeic acid, in black carrots. These findings underscore the potential health benefits associated with the unique composition of phenolic compounds in black carrots and *β*-carotene in orange carrots.

## 4 Conclusions

This study investigated the impact of root colours (red, orange, and black) of different carrot varieties on the quantification of *β*-carotene and other bioactive phytoconstituents. UV–Visible spectrophotometric analysis revealed the highest polyphenol content in black carrots compared to red and orange varieties. UV spectrum analysis further demonstrated that orange carrots exhibit strong UV absorption and contain the highest concentration of carotenoids, particularly *β*-carotene, among the carrot varieties. In contrast, HPLC analysis identified black carrots as having the highest levels of health-promoting phytoconstituents. Chlorogenic acid was found abundantly in all carrot varieties, while additional phenolic compounds were uniquely detected in black carrots. Notably, this study also identified methyl caffeate for the first time in black carrots. Given the well-established health benefits of carrots, all selected varieties emerge as significant sources of nutraceutically rich constituents.

## Funding statement

This research did not receive any external funding. The present work has been conducted using internal research funds from Patanjali Research Foundation Trust, Haridwar, India.

## Data availability statement

The data that support the findings of this study are available from the corresponding author upon reasonable request.

## CRediT authorship contribution statement

**Acharya Balkrishna:** Resources, Project administration, Funding acquisition, Conceptualization. **Monali Joshi:** Visualization, Software, Methodology, Formal analysis. **Sarika Gupta:** Visualization, Software, Methodology, Formal analysis. **M. Priya Rani:** Writing – review & editing, Writing – original draft, Investigation, Formal analysis, Data curation. **Jyotish Srivastava:** Writing – review & editing, Investigation. **Pardeep Nain:** Writing – review & editing, Investigation. **Anurag Varshney:** Writing – review & editing, Supervision, Project administration, Conceptualization.

## Declaration of competing interest

The authors declare that they have no known competing financial interests or personal relationships that could have appeared to influence the work reported in this paper.
